# Sustainable production of bio-crude oil via hydrothermal liquefaction of symbiotically grown biomass of microalgae-bacteria coupled with effective wastewater treatment

**DOI:** 10.1038/s41598-019-51315-5

**Published:** 2019-10-18

**Authors:** Gargi Goswami, Bidhu Bhusan Makut, Debasish Das

**Affiliations:** 1 Department of Biosciences & Bioengineering, Indian Institute of Technology, Guwahati, Assam 781039 India; 2 Center for Energy, Indian Institute of Technology, Guwahati, Assam 781039 India

**Keywords:** Biotechnology, Bioremediation

## Abstract

The study demonstrates a sustainable process for production of bio-crude oil via hydrothermal liquefaction of microbial biomass generated through co-cultivation of microalgae and bacteria coupled with wastewater remediation. Biomass concentration and wastewater treatment efficiency of a tertiary consortium (two microalgae and two bacteria) was evaluated on four different wastewater samples. Total biomass concentration, total nitrogen and COD removal efficiency was found to be 3.17 g L^−1^, 99.95% and 95.16% respectively when consortium was grown using paper industry wastewater in a photobioreactor under batch mode. Biomass concentration was enhanced to 4.1 g L^−1^ through intermittent feeding of nitrogen source and phosphate. GC-MS and FTIR analysis of bio-crude oil indicates abundance of the hydrocarbon fraction and in turn, better oil quality. Maximum distillate fraction of 30.62% lies within the boiling point range of 200–300 °C depicting suitability of the bio-crude oil for conversion into diesel oil, jet fuel and fuel for stoves.

## Introduction

Unfettered exploitation of the fossil fuels integrated with unimpeded population growth and brisk industrialization are the root causes of the escalating world energy crisis and intensifying global warming by and large. Global disquietude for energy security and environmental sustainability has propelled the scientific community to contemplate conceivable energy resources as prospective substitutes. Presently, several countries across the globe are capitalizing on biomass-, wastes-, solar-, wind-, hydro- and geothermal energy sources, with net-zero carbon emissions, as futuristic alternatives to fossil based fuels. Microalgal biofuel has been gaining impetus owing to their several advantages such as high photosynthetic efficiency coupled with sequestration of carbon dioxide, high growth rate, momentous capability to accumulate lipids/carbohydrates and significant tolerance towards manifold cultivation conditions. An auxiliary characteristic is the wide paradigm of value-added bio-products which can be obtained from these organisms, besides the fuel counterpart. Though the concept of biofuel production using microalgal feedstock is gaining ground, yet certain challenges pose a major hindrance towards commercialization of this scientific know-how. The high production cost of microalgal biomass using fresh water coupled with energy and cost intensive downstream processing constitute the dominant threat towards the economic feasibility of this technology.

A realistic solution to the techno-economic infeasibility of microalgal biofuel technology can be the concept of co-cultivation of microalgae and bacteria in wastewater as an integrated system. Several studies have been reported in the literatures on production of biofuels using mixed-culture algal biomass grown in wastewater^1,2^. However, in recent years microalgae-bacteria consortia have been gaining impetus in generation of biomass feedstock coupled with wastewater treatment. This may be attributed to various advantages which microalgae-bacteria consortia might offer over mixed-culture algal system: (i) ability to function under fluctuating environmental conditions and nutrient loading owing to their diverse metabolic activities and adaptation ability to different growth conditions; (ii) microalgae and bacteria can form micro-ecosystem where they can positively influence the growth of each other in different ways^3^; and (iii) certain bacteria can facilitate the harvesting ability of algal cells^4^. There are several studies which have reported the enhanced growth of microalgae when co-cultured with bacteria^5,6^. Wastewater is a convenient source of nitrogen, phosphorous and micronutrients, essential for the growth of microalgae, besides being a reasonable replacement of fresh water. Microalgae have been reported to possess noteworthy potential to utilize inorganic nutrients, copious in wastewater, to produce biofuels, fertilizers or other bio products supplementing the wastewater treatment^7,8^. Microalgae cultivation in wastewater therefore offers a two-fold advantage: (i) minimizes the cost of biomass production and (ii) aids in wastewater remediation. An accretion in nutrient removal efficiency from wastewater was reported when microalgae were grown in consortium with bacteria^9,10^. This improved biomass concentration and nutrient removal efficiency may be attributed to the concerted interplay between microalgae and bacteria within their micro-ecosystem through various means of interaction *e.g*., exchange of gases or growth promoting factors, reduction of photosynthetic oxygen tension, bacterial decomposition of dissolved organic nitrogen etc.^6,8,11–13^.

High cost of downstream processing is another prime drawback towards sustainable biofuel production using microalgal biomass feedstock. Transesterification, the traditional method for producing biodiesel from lipid rich microalgal biomass is energy intensive as it requires biomass drying; expensive; time consuming; and employs hazardous organic solvents. Moreover, post transesterification the residual major fraction of the biomass, comprising of protein and carbohydrate, remain unutilized and hence, the overall process efficiency is compromised. In order to address the existing challenges as imposed by the traditional transesterification method, researchers are now focusing on hydrothermal liquefaction (HTL) of biomass into biofuel. The key advantage of HTL includes direct conversion of wet algal biomass (~80% water content, w/w) into bio-crude oil and hence, bypassing the need for drying. Unlike transesterification, where only lipid fraction of the microalgal biomass is converted into biofuel, HTL facilitates conversion of the entire fraction of biomass (lipids, proteins and carbohydrates) into bio-crude oil^14^. Further, microbial consortium including bacteria, wastewater sludge and fast growing algae with low lipid content can successfully be converted into biofuel via HTL^15^.

The present study demonstrates a sustainable process for production of biofuel using microbial biomass as potential feedstock. Sustainability was aimed to be achieved through a combinatorial approach of: (i) cultivation of a microbial consortium comprising of two microalgae and two bacteria; (ii) utilization of wastewater as a cheaper source of nutrients and replacement of fresh water; (iii) improving biomass productivity through intermittent feeding of limiting nutrients and mutualistic growth of the microbes; (iv) direct conversion of wet biomass into bio-crude oil and (v) biomass generation coupled with wastewater treatment. In an earlier study, a tertiary consortium comprising of two microalgae and two bacteria was shown to exhibit superior performance in terms of biomass concentration and nutrient removal efficiency from the artificial wastewater and raw dairy wastewater^16^. In the first step, with the aim of establishing the feasibility of application at industrial scale, performance of this tertiary consortium in terms of biomass concentration and wastewater treatment efficiency was evaluated on four different types of wastewater: paper industry wastewater (PWW), textile industry wastewater (TWW), leather industry wastewater (LWW) and municipal wastewater (MWW). Based on the microbial biomass concentration and nutrient removal efficiency obtained in different wastewaters, PWW was selected for subsequent experimentation. In the next step, with the objective of maximization of microbial biomass concentration, a process engineering strategy was implemented in an automated photobioreactor involving intermittent feeding of the limiting nutrients under fed-batch mode of cultivation of the consortium on PWW. The wet microbial biomass thus obtained from fed-batch cultivation was further subjected to HTL for direct conversion into bio-crude oil. Extensive characterization of the bio-crude oil was carried out to assess its potential as an alternative to conventional fossil fuels.

## Results and Discussion

### Characterization of tertiary consortium on different wastewater samples

With the aim of assessing its potential to be exploited at commercial scale, the tertiary consortium has been characterized in terms of its growth and wastewater treatment efficiency on different wastewater samples. Four different types of wastewater were considered in the present study: PWW, TWW, LWW and MWW. These wastewaters differed in terms of concentration of the nutrients and pH. Major nutrients such as total nitrogen, phosphate and COD content were found to be abundant in case of PWW and MWW (Table 1). However, these nutrients were present in lesser amount in case of TWW and LWW. All the wastewater samples contained significant amount of heavy metals, chromium and nickel, with the exception of MWW (Table 1).

**Table 1:** Analysis of wastewater samples.

Wastewater Type	Parameters								
	pH	Total Nitrogen (mg L^−1^)	NO_3_^−^-N (mg L^−1^)	NO_2_^−^-N (mg L^−1^)	NH_4_^+^-N (mg L^−1^)	Phosphate (mg L^−1^)	COD (mg L^−1^)	Chromium (mg L^−1^)	Nickel (mg L^−1^)
Paper Industry	6.29 ± 0.09	490 ± 0.02	187 ± 0.02	0	303 ± 0.02	77.70 ± 0.073	5250.0 ± 0.27	0.98 ± 0.02	0.53 ± 0.04
Textile Industry	7.74 ± 0.01	230 ± 0.07	110 ± 0.04	0.29 ± 0.14	118.71 ± 0.03	53.16 ± 0.012	735.00 ± 0.31	0.27 ± 0.02	0.43 ± 0.07
Leather Industry	7.53 ± 0.04	250 ± 0.04	162 ± 0.03	0.1 ± 0.03	89.9 ± 0.06	48.30 ± 0.061	400.50 ± 0.14	0.69 ± 0.06	1.01 ± 0.03
Municipal Wastewater	7.42 ± 0.04	540 ± 0.02	112 ± 0.01	0	428 ± 0.03	69.20 ± 0.049	1650.00 ± 0.25	*ND	*ND

*ND: Not Detected.

**Figure 1:**
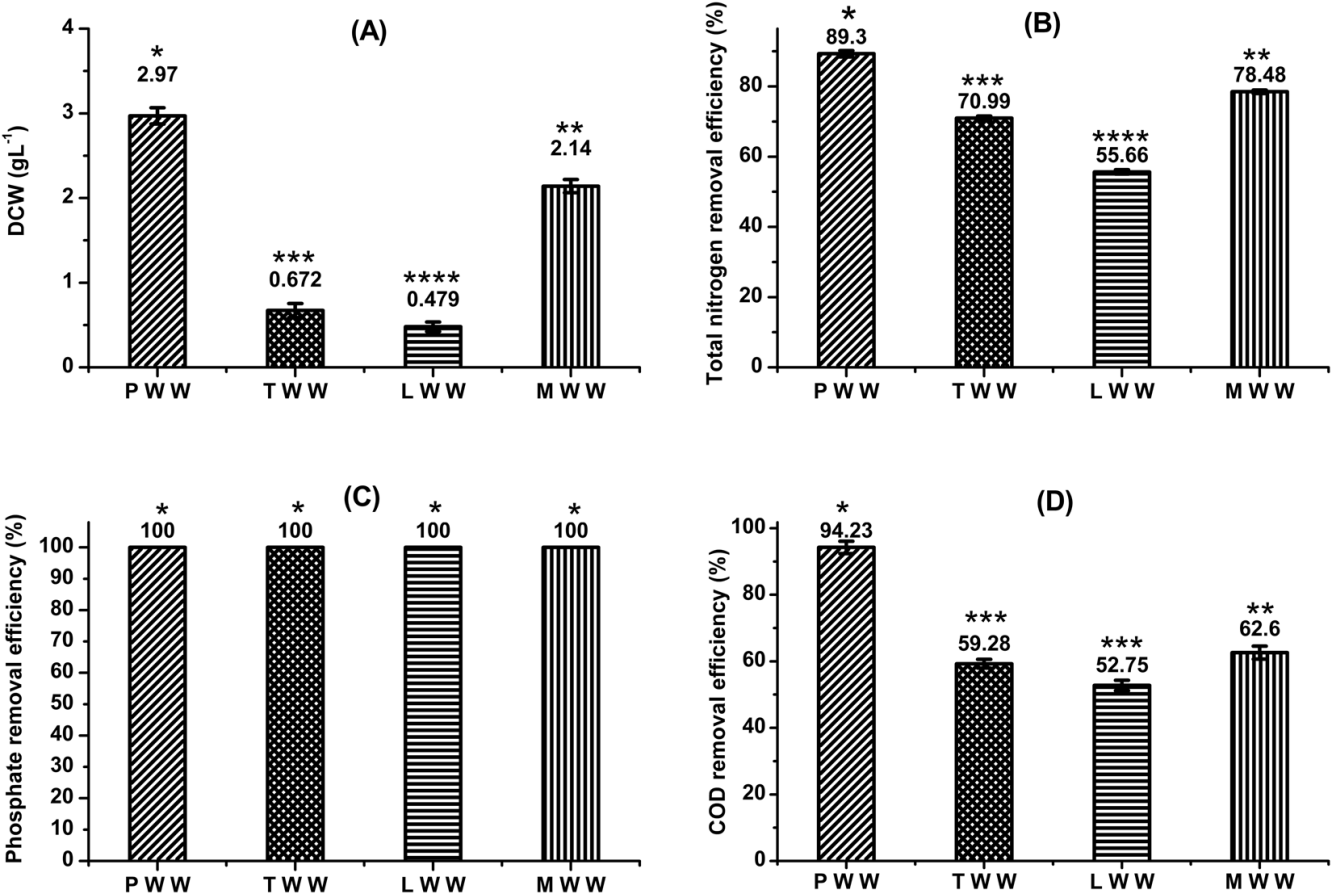
(**A**) Total biomass concentration (DCW, g L^−1^), (**B**) total nitrogen removal efficiency (%), (**C**) phosphate removal efficiency (%) and (D) COD removal efficiency (%) of the tertiary consortium grown on paper industry wastewater (PWW), textile industry wastewater (TWW), leather industry wastewater (LWW) and municipal wastewater (MWW). The *asterisk sign* represents the significant difference between the biomass concentration or nitrate removal efficiency or phosphate removal efficiency or COD removal efficiency obtained for different wastewater samples analyzed using one-way analysis of variance based on Tukey’s method. Biomass concentration or nitrate removal efficiency or phosphate removal efficiency or COD removal efficiency that do not share a common symbol are significantly different.

Figure 1 elucidates the comparative performance of the tertiary consortium in different wastewater samples with respect to growth and removal efficiency (%) of total nitrogen, phosphate and COD. Maximum total microbial biomass concentration of 2.97 g L^−1^ was achieved when tertiary consortium was grown in PWW, followed by 2.14 g L^−1^ in MWW. Growth performance of the consortium was inferior in both LWW and TWW (Fig. 1A). Further, an improved microalgal growth was observed in case of PWW and MWW with a final chlorophyll-a concentration of 31.79 µg mL^−1^ (10.7 µg mg biomass^−1^) and 21.87 µg mL^−1^ (10.22 µg mg biomass^−1^) respectively (see Supplementary Fig. S1). In spite of the wide variation in the initial concentrations of total nitrogen, phosphate and COD, these nutrients were exhausted within similar cultivation period for all wastewater samples (see Supplementary Fig. S1), depicting higher utilization rates of these nutrients by the microbes when grown on PWW and MWW and in turn higher growth rate of the microbes. A biomass concentration of 826 mg L^−1^ was reported when *C. vulgaris* was co-cultivated with indigenous bacteria in municipal wastewater^17^. Das *et al*.^18^ has reported a chlorophyll-a concentration of 12.5 µg mL^−1^ when a marine species of *Chlorella* was grown in consortium with *Phormidium* sp. in tannery wastewater. Better growth performance of the consortium in PWW and MWW may be attributed to higher amount of inorganic nutrients (total nitrogen and phosphate) primarily supporting growth of the microalgae^19,20^ and higher amount of COD primarily favoring growth of the bacteria^21^ in comparison to TWW and LWW. Microalgae assimilates inorganic nitrogen sources into organic macromolecules and genetic materials and hence, critically essential for their growth^22^. Phosphorous also play crucial role in microalgal energy metabolism via synthesis of nucleic acids, lipids, proteins and the intermediates of carbohydrate^23^. In a microalgae-bacteria consortium, bacterial strains remove COD through aerobic biological treatment involving oxidative degradation of the organic compounds for both energy and carbon usages^24^.

This improved growth performance of the consortium in PWW and MWW correlated well with the removal efficiency of the key nutrients *viz*., total nitrogen, phosphate and COD. The highest nitrogen removal efficiency of 89.3% was observed in case of PWW followed by MWW (78.48%) and TWW (70.99%) (Fig. 1B). The nitrogen removal efficiency was the lowest at a value of 55.66% when grown on LWW. Phosphate was completely exhausted for all wastewater samples as seen in Fig. 1C. While, COD removal efficiency was maximum at a considerably higher value of 94.23% in case of PWW, the same was merely in the range of 52–62% for rest of the wastewater samples (Fig. 1D). Total nitrogen and phosphate removal efficiency of 95.9% and 94.4% respectively was achieved from cultivation of *C. vulgaris* with indigenous bacteria in municipal wastewater^17^. An algae-bacteria combined system of *C. vulgaris* and *Bacillus licheniformis* when grown in municipal wastewater resulted in removal of ammoniacal nitrogen and phosphate with an efficiency of 78% and 92% respectively^25^. Bioremediation of tannery wastewater by a consortium of marine *Chlorella* sp. and *Phormidium* sp. resulted in 91.16% nitrogen and 88.88% phosphate removal^18^. COD removal efficiency of ~91% was achieved when microalgae-bacteria consortia was cultivated in wastewater from potato processing plant^21^, dairy industry^26^ and municipality^27^. While, a commendable removal efficiency of both the heavy metals (61.95% for Ni and 45.71% for Cr) was observed in PWW, the same was lower in LWW (Table 2). However, an effective Cr removal (56.38%) was observed in case of TWW. Das *et al*.^18^ has reported 94.45% removal of Cr from tannery wastewater employing a consortium of *Chlorella* sp. and *Phormidium* sp. Based on the maximum total biomass concentration and nutrient removal efficiency, PWW was selected for subsequent characterization and process development in the bioreactor. A detailed material balance for total nitrogen, phosphate and COD has been shown in Supplementary Table S1.

**Table 2:** Heavy metal removal efficiency (%) of tertiary consortium grown in different types of wastewater.

Wastewater Type	Nickel (Ni)	Chromium (Cr)
Shake flask experiments		
Paper Industry	61.95 ± 0.07	45.71 ± 0.09
Textile Industry	30.00 ± 0.04	56.38 ± 0.02
Leather Industry	35.50 ± 0.05	22.07 ± 0.04
Municipal Wastewater	*ND	*ND
Bioreactor		
Paper Industry	74.54 ± 0.04	80.00 ± 0.07

*ND: Not Detected.

All the experiments were performed in duplicate and the data were expressed as mean ± standard error.

### Batch and fed-batch mode of cultivation of tertiary consortium in the bioreactor utilizing paper industry wastewater

**Figure 2:**
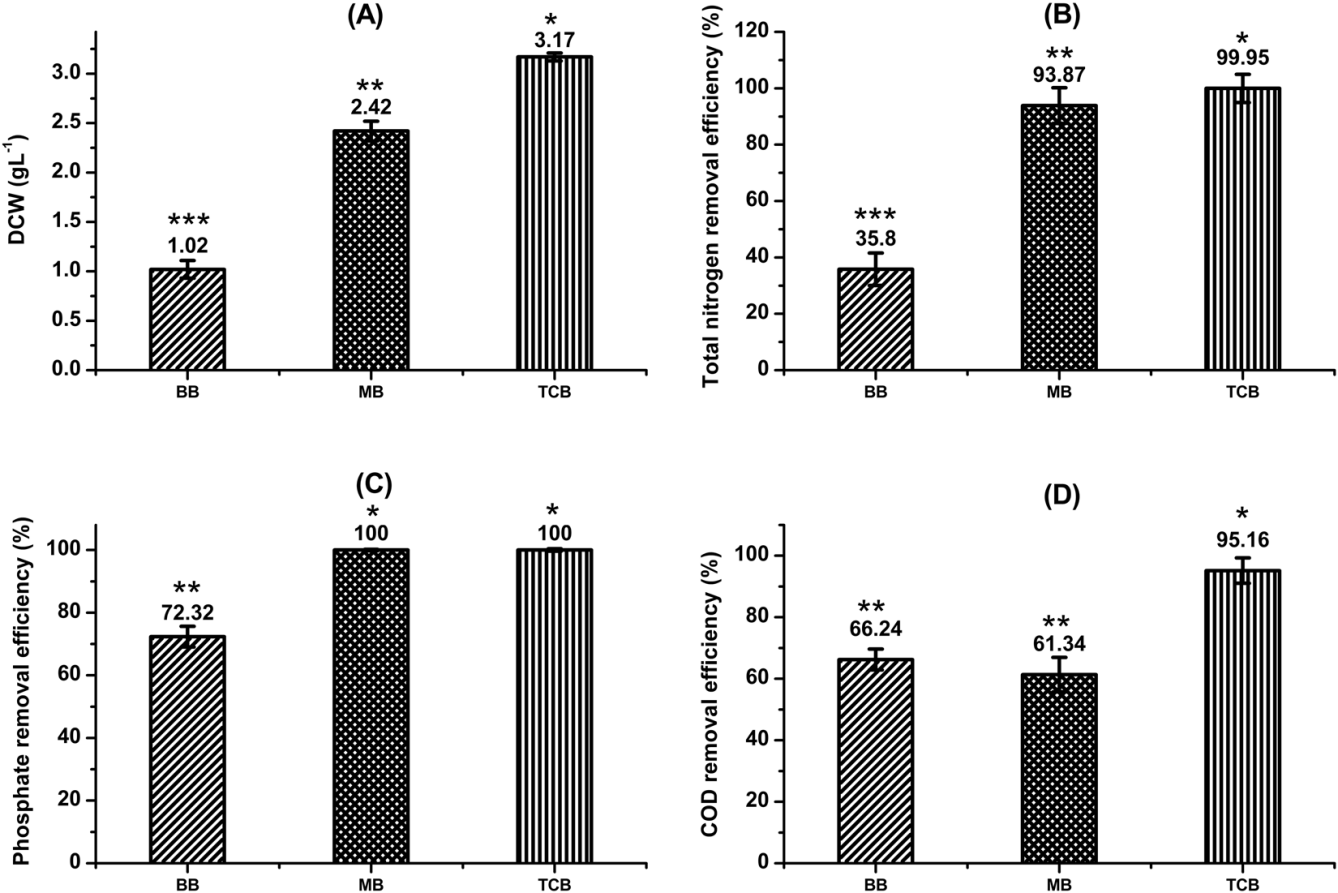
(**A**) Total biomass concentration (DCW, g L^−1^), (**B**) total nitrogen removal efficiency (%), (**C**) phosphate removal efficiency (%) and (D) COD removal efficiency (%) of the tertiary consortium grown on paper industry wastewater in photobioreactor under batch mode of cultivation. BB represents bacterial batch involving two bacteria; MB represents microalgal batch involving two microalgae and TCB represents tertiary consortium batch involving two microalgae and two bacteria. The *asterisk sign* represents the significant difference between the biomass concentration or nitrate removal efficiency or phosphate removal efficiency or COD removal efficiency obtained for BB, MB and TCB analyzed using one-way analysis of variance based on Tukey’s method. Biomass concentration or nitrate removal efficiency or phosphate removal efficiency or COD removal efficiency that do not share a common symbol are significantly different.

In order to evaluate the performance of the tertiary consortium in PWW at a larger scale of operation, characterization was carried out in 7.5 L automated bioreactor with a working volume of 4 L. A condescending performance in terms of growth and nutrient removal efficiency was exhibited by the tertiary consortium (TCB) in comparison with bacteria (BB) and microalgae (MB) when cultivated individually in batch mode of operation (Fig. 2). For instance, an increment in total biomass concentration of 211% and 31% was achieved for the TCB (3.17 g L^−1^) compared to BB and MB respectively (Fig. 2A). For TCB, the final chlorophyll-a concentration was evaluated to be 32.50 µg mL^−1^ which was 1.2 folds higher than the chlorophyll-a concentration obtained (27.30 µg mL^−1^) in the MB. However, the normalized chlorophyll-a concentration was evaluated to be 10.25 µg mg biomass^−1^ and 11.28 µg mg biomass^−1^ for TCB and MB respectively. Cho *et al*.^28^ reported a biomass concentration of 3.31 g L^−1^ when *C. vulgaris* was co-cultivated with four different growth enhancing bacteria. However, removal efficiency for total nitrogen was found to be analogous for both TCB (99.95%) and MB (93.87%), albeit a reduction by 2.7 folds in case of BB (35.8%) (Fig. 2B). Similar trend was observed in case of phosphate removal. While, phosphate was completely exhausted for both TCB and MB, 72.32% removal was observed in case of BB (Fig. 2C). Interestingly, an uppermost COD removal by 95.16% was recorded for TCB, significantly higher than both MB and BB (Fig. 2D). The total nitrogen, phosphate and COD removal efficiency of TCB, achieved in the present study, was found to be better than majority of literatures reported till date. For instance, phosphate and nitrogen removal efficiency of 47% and 62% respectively was reported for co-cultivation of immobilized *C. sorokiniana* and *Azospirillum brasilense* on unsterile municipal wastewater^29^. Phosphate, total nitrogen and COD removal efficiency of 72.8%, 71% and 46% respectively was achieved for combined growth of *C. sorokiniana* and *Pseudomonas* H4 in wastewater^30^. However, a complete removal of both nitrogen and phosphate along with 97% removal of COD was observed on co-cultivation of *C. vulgaris* FACHB-30 and *P. putida* in municipal wastewater^31^. Removal efficiency of Ni and Cr was observed to be significantly enhanced to 74.54% and 80% respectively as compared to growth in shake flasks utilizing PWW (Table 2).

**Figure 3:**
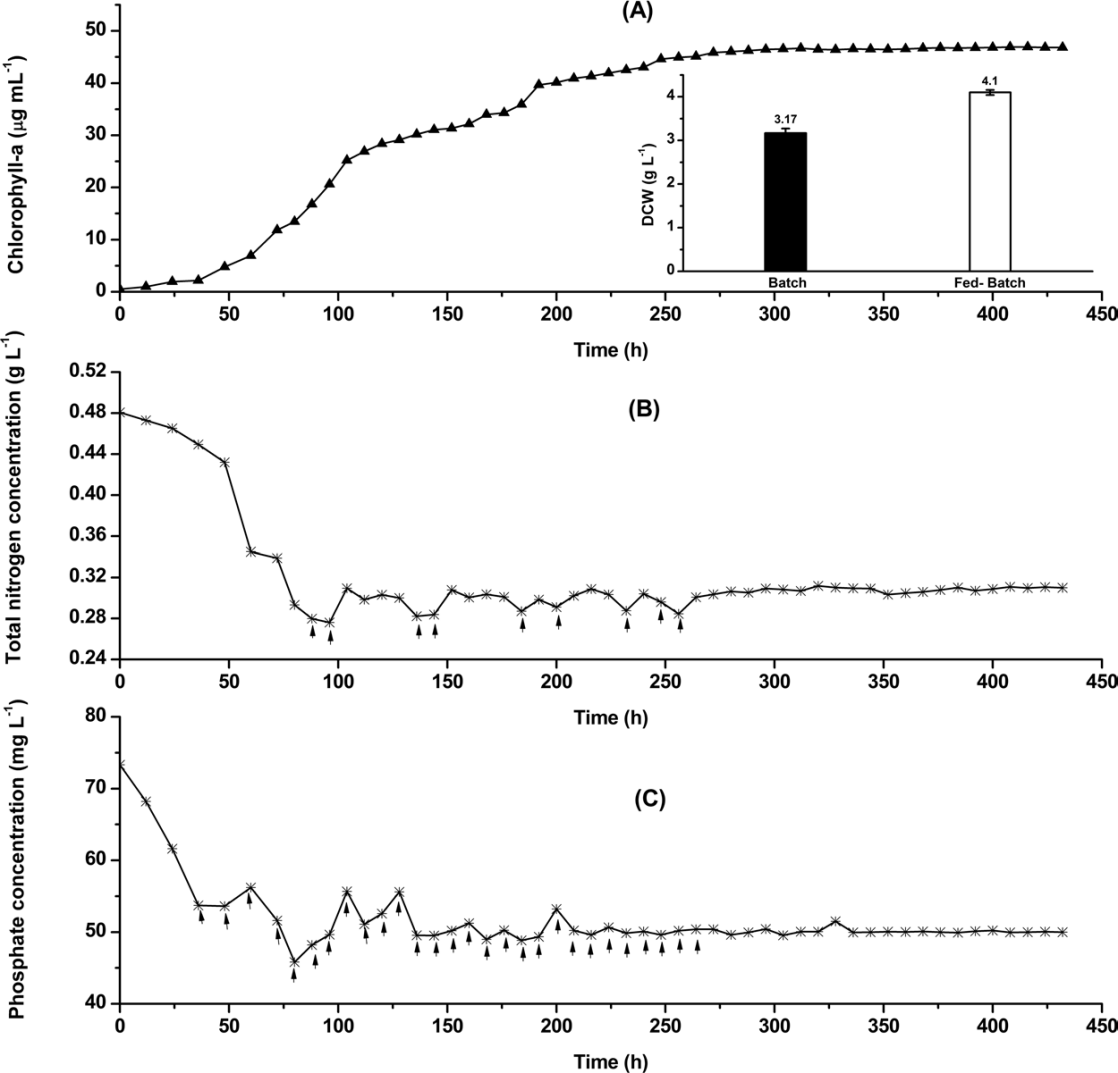
Dynamic profiles for (**A**) growth of microalgae (Chlorophyll-a, µg mL^−1^); (**B**) total nitrogen (g L^−1^) and **(C**) phosphate (mg L^−1^). The tertiary consortium was grown on paper industry wastewater in a photobioreactor under fed-batch mode with intermittent feeding of nitrogen source and phosphate. The graph in the inset of panel A compares total biomass concentration obtained in batch and fed-batch cultivation.

With the aim of improving the biomass concentration, cultivation of tertiary consortium was conducted through intermittent feeding of nitrogen source and phosphate in order to maintain their respective optimal concentrations throughout the entire course of fermentation. As observed from the figure (Fig. 3B,C), concentration of total nitrogen and phosphate in the culture broth was maintained at approximately 0.29 g L^−1^ and 49.5 mg L^−1^ respectively. Total biomass concentration was evaluated to be 4.1 g L^−1^, an increment by 29.3% when compared to the batch mode of cultivation (Fig. 3A). A total biomass concentration in the range of 2.23–3.15 g L^−1^ was obtained when cyanobacterium *Arthrospira platensis* and microalga *C. vulgaris* were cultivated in fed-batch mode in wastewater derived from the anaerobic digestion of poultry litter depending on different concentrations of ammoniacal nitrogen^32^. The maximum concentration of chlorophyll-a was observed to be 46.92 µg mL^−1^ (11.44 µg mg biomass^−1^) when the tertiary consortium was cultivated in PWW in fed-batch mode of operation. This chlorophyll-a concentration was found to be 1.44 times higher than that obtained in batch mode.

### Symbiotic growth of microalgae and bacteria

**Figure 4:**
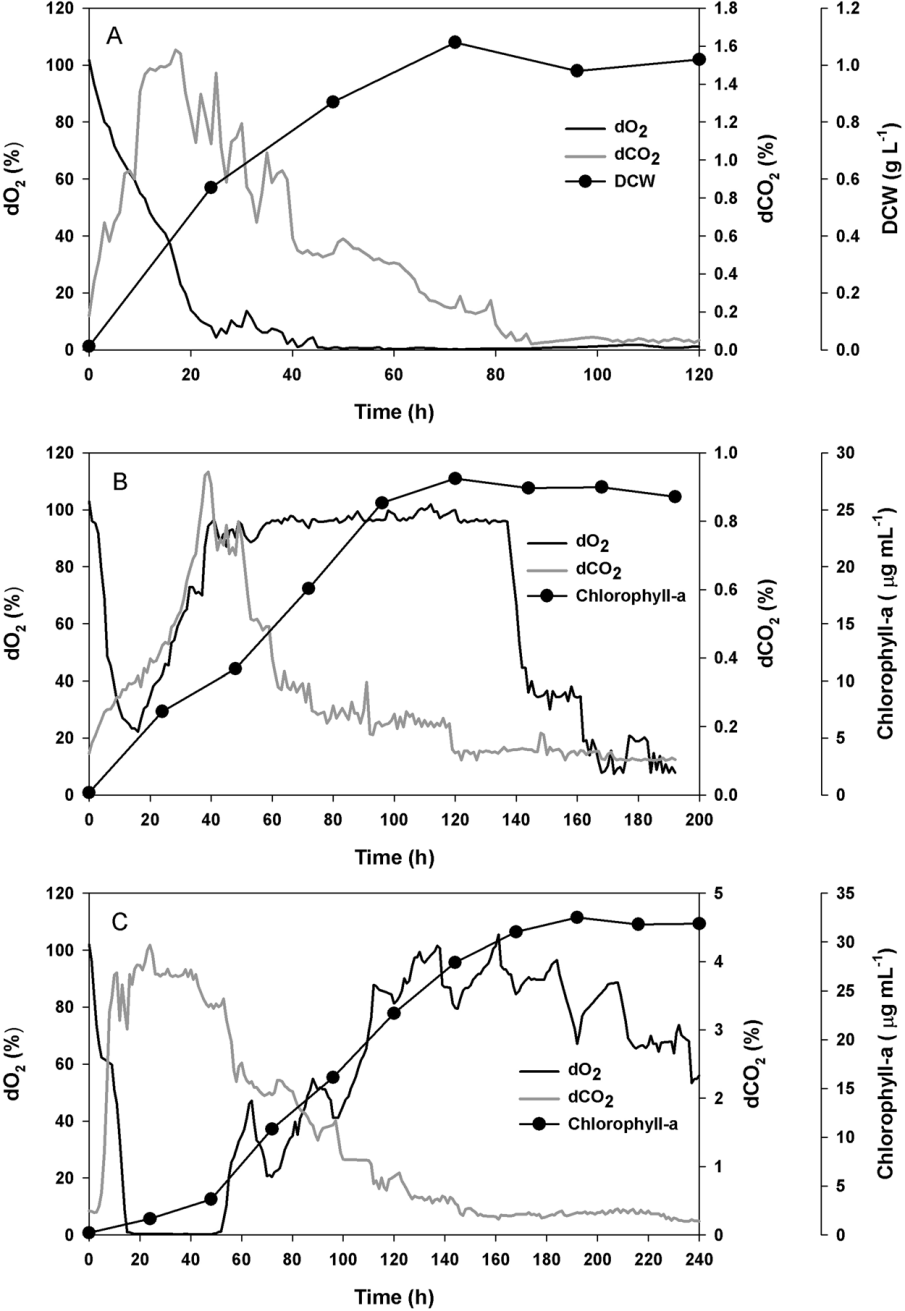
Dynamic profiles for: (**A**) dO_2_ (%), dCO_2_ (%) & growth of bacteria (DCW, g L^−1^) in case of bacterial batch (BB); (**B**) dO_2_ (%), dCO_2_ (%) & growth of microalgae (Chlorophyll-a, µg mL^−1^) in case of microalgal batch (MB); and (**C**) dO_2_ (%), dCO_2_ (%) & growth of microalgae (Chlorophyll-a, µg mL^−1^) in case of tertiary consortium batch (TCB). The experiment was carried out in a photobioreactor under batch mode of cultivation.

In the present study, an enhancement in total biomass concentration and in turn, nutrient removal efficiency was observed when a consortium of two microalgae and two bacteria were grown together (TCB) as compared to growth of two bacteria (BB) or two microalgae (MB) alone. The possible reasons for this improved biomass concentration and nutrient removal efficiency may be attributed to various mutualistic interactions between microalgae and bacteria *e.g*., symbiotic exchange of CO_2_ and O_2_^13^ and supply of micronutrients and growth promoting factors from bacteria^28,33,34^. An inhibition in the growth of the bacteria in BB was expected post 40 h of cultivation as the dO_2_ level dipped significantly below saturation value and in turn, resulted in limitation to the oxygen supply (Fig. 4A). However, in this batch the peak dCO_2_ concentration was recorded to be ~1.5% during exponential phase of the bacterial growth (Fig. 4A). Contrary to the BB, in the MB an initial drop in the dO_2_ level was marked till 20 h followed by a sharp increase and its maintenance close to the saturation value till ~140 h (Fig. 4B). While the initial drop in the dO_2_ level may be due to the consumption of oxygen by the native bacteria present in the wastewater, increase in the dO_2_ level can be attributed to the initiation of exponential growth of the microalgae after 20 h which continued till 120 h (Fig. 4B). A concomitant drop in dO_2_ level was observed with the attainment of stationary phase of microalgal growth. Nevertheless, in this batch, rate of CO_2_ supply appeared to be the rate controlling step as evident from lower dCO_2_ level (maximum of <1%) as compared to the BB. Interestingly, in case of TCB while dO_2_ level above 40% was maintained for entire cultivation period except the initial hours where a sudden decrease in the dO_2_ level was due to combined growth of the native bacteria present in the wastewater and the bacteria present in the consortium (Fig. 4C). The maintenance of dO_2_ level above 40% post 50 h of cultivation corroborated well with the growth of microalgae contributing to continuous release of oxygen in the culture broth (Fig. 4C). A higher dissolved carbon dioxide tension in the range of 1–4% was maintained in TCB for a prolonged cultivation period of 120 h, which might be attributed to the sustained growth of the bacteria in the consortium (Fig. 4C). While the bacteria consumed oxygen produced by microalgae during photosynthesis as an electron acceptor to degrade organic matter (COD) present in the wastewater, the microalgae fixed CO_2_ released as by-product of bacterial oxidative degradation. This mutualistic interaction between microalgae and bacteria in terms of exchange of CO_2_ and O_2_ promoted the individual growth of these organisms present in the tertiary consortium, which resulted in improved total biomass concentration and better nutrient removal efficiency. In a previous study, glucose removal efficiency was reported to increase from 50% to 100% in batch mode of operation and 73% to 100% in continuous mode of operation of the bioreactor when immobilized microalgae *C. vulgaris* was co-cultivated with *P. putida*^13^. This improvement in glucose utilization was attributed to a symbiotic CO_2_/O_2_ gas exchange between these two microbes. Further, an exponential growth of the microalgae was observed during the symbiosis when compared to unaerated condition^13^. However, in the present study existence of any other possible interaction may not be ruled out for growth upliftment.

### Characterization of bio-crude oil produced via hydrothermal liquefaction of microbial biomass

The yield of bio-crude oil obtained from HTL of microbial biomass generated by growing tertiary consortium in PWW was 15% (w/w). The bio-crude oil yield obtained for TCB was found to be significantly higher than BB (4.4%, w/w) and MB (8.2%, w/w). This better bio-crude oil yield in case of TCB might be due to difference in composition of biomass feedstock as compared to BB and MB. However, the bio-crude oil yield obtained from TCB of the present study was found to be 41.69% and 39.02% lower than that reported for mixed culture of microalgae^1^ or microalgae-bacteria consortia^35^, respectively. This inferior yield might be due to the un-optimized process parameters and quality of the biomass feedstock. The lower yield of biocrude-oil from TCB can be improved by optimizing the HTL parameters e.g., biomass loading, residence time and reaction temperature^36^. Further, *in-situ* catalytic treatment is expected to increase the bio-crude oil yield^37^. Elemental composition of the bio-crude oil in terms of C, H, N, S and O (%) was found to be 63.16, 8.11, 5.37, 0.21 and 23.15 respectively. This elemental composition of the bio-crude oil obtained in the present study was found to be comparable with the biocrude obtained via HTL of other microalgae^38^. However, heavy metals and inorganic phosphorous was not found to be present in the bio-crude oil.

**Figure 5:**
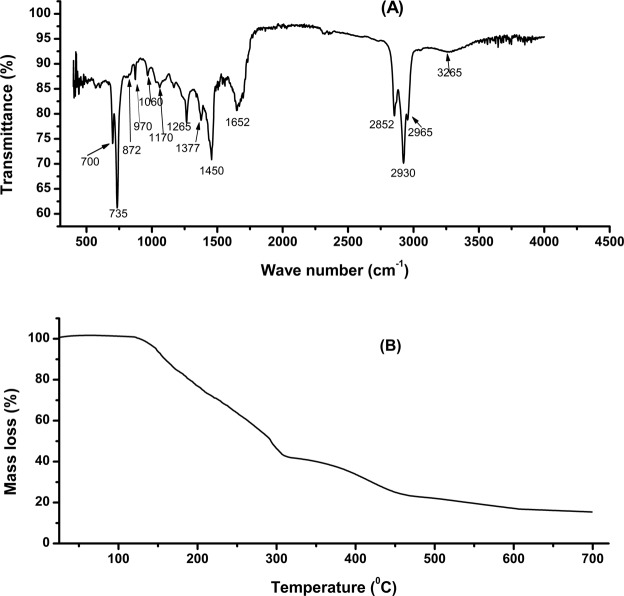
(**A**) FTIR spectra and (**B**) TGA curve of bio-crude oil obtained via hydrothermal liquefaction of microbial biomass.

The FT-IR spectra of bio-crude oil exhibited characteristic absorption peaks reflecting presence of various functional groups and in turn, corresponding compounds (Fig. 5A). The band at 3265 cm^−1^ refers to the O-H stretching indicating presence of water or alcohol in the bio-crude oil. The bands at 2965, 2930, 2852, 1450 and 1377 cm^−1^ depict C-H vibrations, indicating the presence of alkanes. The C=O stretching vibration at 1652 cm^−1^ attributed to the presence of ketones, aldehydes and carboxylic acids. C-O stretching at 1265, 1170, 1060 and 970 cm^−1^ represents presence of primary, secondary and tertiary alcohols in the bio-crude oil. Existence of phenols, esters, ethers and aromatic compounds are represented by the presence of O-H bends at 872, 735 and 700 cm^−1^. These results corroborate with the findings reported by Shuping *et al*.^39^.

GC-MS analysis (Table 3) revealed that more than 82% of the bio-crude oil was composed of hydrocarbons, alcohols, amines, amides, aldehydes and ketones. These findings complement the results obtained from FTIR analysis. The list of all major compounds with a score of 75 on a scale of 100 have been enlisted in Table 3. Percentage of hydrocarbon content (1, 2, 4, 6, 7, 8, 11, 12, 13, 14, 17, 18, 19, 20, 25, 26 and 28) in the bio-crude oil was found to be high, depicting good oil quality. The presence of nitrogenous compounds (5, 9 and 10) in bio-crude oil indicates thermal conversion of the protein fraction present in the biomass. The oxygen content in the bio-crude oil may be attributed to the presence of oxygen containing functional groups such as ketones, aldehydes, alcohols and esters produced by the decomposition of proteins and polysaccharide fractions of the microbial biomass. This oxygen and nitrogen content could interfere with the quality of the bio-crude oil and hence, additional up gradation in terms of deoxygenation and denitrogenation is essential prior to its application as fuel oil. The boiling point distribution of the bio-crude oil has been estimated using TGA in nitrogen atmosphere (Fig. 5B). As per the analysis, the maximum distillate fraction of 30.62% lies within the boiling point range of 200–300 °C (Table 4) depicting suitability of the bio-crude oil for conversion into diesel oil, jet fuel and fuel for stoves. It is interesting to note that N, P and chromium was completely absent in HTL aqueous phase with presence of nickel in trace amount (0.04 mg L^−1^). Therefore, this post HTL aqueous phase can be recycled for subsequent cultivation of microalgae or can be used as utility in other industrial applications.

**Table 3:** Major chemical compounds present in the bio-crude oil obtained from hydrothermal liquefaction of tertiary consortium biomass grown in paper industry wastewater.

Sl. No.	Retention time (min)	Name of the compound	Area (%)
1	3.11	Undecane	1.16
2	3.21	Benzene, 1-ethyl-3-(1-methylethyl) -	1.20
3	3.25	Bicyclo [2.2.1] heptan-2-ol, 4-chloro-1, 7, 7-trimethyl-, exo-	1.48
4	7.51	Pyrene, 1, 3-dimethyl-	1.95
5	8.01	benzene acetonitrile, 4, 4′-[1, 2-ethenediyl] bis-	1.51
6	8.73	Tetracosane	1.44
7	9.49	1, 4-Dimethyl-6-phenyl-naphthalene	1.90
8	9.80	Phenanthrene, 2, 3, 5-trimethyl-	3.78
9	10.73	2-Phenazinecarbonitrile, 7-amino-	5.37
10	27.00	Dibenzepin	0.50
11	27.10	Fluoranthene	1.39
12	27.39	n-Heptadecene	0.99
13	27.59	Octadecane, 2, 6, 10, 14-tetramethyl-	0.49
14	27.70	Phenanthrene, 4, 5-dimethyl-	0.61
15	27.84	10-Hydroxynortriptyline	10.72
16	28.18	3, 7-Dimethyldibenzothiophene	1.92
17	28.44	5-Methoxy (5 H) dibenzo [a, d] cycloheptene	0.57
18	28.65	Bicyclo [2.2.1] heptane, 3-methylene-2-(3-phenylprop-1-en2yl)-	10.55
19	28.69	Naphthalene, 1-(phenylmethyl) -	2.60
20	28.76	Phenanthrene, 3, 6-dimethyl-	5.80
21	29.18	3-(3-Indolyl)−5-oxo-3-pyrazoline-4-carbonitrile	0.56
22	29.26	Normethadone	3.05
23	29.37	Benzo[b]naphtha [2, 3-d] thiophene, 7, 8, 9, 10-tetrahydro-	2.16
24	30.03	3-Naphthalen-2-yl-3-piperidin-1-yl-propan-1-ol	2.09
25	30.18	Phenanthrene, 3, 6-dimethyl	0.61
26	30.45	Phenanthrene, 2, 5-dimethyl-	3.82
27	30.67	Isobenzofuran, 1, 3-dihydro-1, 1-dimethyl-3-phenyl-	0.83
28	30.92	Pyrene, 4, 5, 9, 10-tetrahydro	3.20
29	31.08	4-(1-Methyl-1-siletanyl) phenol	9.90
30	31.61	Benzo[b]selenophene-3-carboxaldehyde, 2-methyl-	0.50
Total area (%)			82.65

**Table 4:** Distillate range of different fractions of bio-crude oil obtained via thermogravimetric analysis.

Distillate Range (°C)	Typical Applications	Fraction of bio-crude oil (%)
25–100	Bottle gas and chemicals	2.33
100–200	Gasoline	23.20
200–300	Jet fuel, fuel for stoves and diesel oil	30.62
300–400	Lubricating oil for engines, fuel for ships and machines	12.51
400–550	Lubricants and candles	14.11
550–700	Fuel for ships and factories	2.60

## Conclusions

A sustainable process was demonstrated towards production of bio-crude oil from microbial biomass feedstock via combinatorial approach of: (i) improved biomass concentration through intermittent feeding of limiting nutrients and mutualistic growth of microalgae-bacteria; (ii) utilization of wastewater as cheap source of nutrients and water; (iii) direct conversion of wet biomass into bio-crude oil via hydrothermal liquefaction and (iv) simultaneous remediation of wastewater. The process may be a potential technology platform towards sustainable production of bio-crude oil from microbial biomass utilizing wastewater.

## Methods

### Sampling and characterization of different wastewaters

Four different types of wastewater samples were collected from effluent treatment plants of the Guwahati Municipal Corporation (GMC) and different industries across Guwahati (26.1445 °N, 91.7362 °E), Assam, India. These consisted of samples from (i) paper industry, (ii) textile industry, (iii) leather industry and (iv) GMC. The wastewater samples were allowed to settle down in order to eliminate the interference of total suspended particles. Subsequent to physical separation of the suspended particles, the pH, total nitrogen, phosphorus, chemical oxygen demand (COD) and concentrations of two heavy metals, namely Chromium (Cr) and Nickel (Ni) present in the wastewater samples, were measured.

### Microalgae and bacteria consortium

In a previous study, Makut *et al*.^16^ designed a tertiary consortium comprising of two microalgae and two bacteria towards generation of biomass feedstock utilizing wastewater as cheaper source of nutrients. These organisms were isolated from oil refinery wastewater sample collected from Indian Oil Corporation Limited, Noonmati (26.19 °N, 91.80 °E), Guwahati, Assam, India. The microalgae were identified as *Chlorella sorokiniana* strain DBWC2 & *Chlorella* sp. strain DBWC7 and the bacteria were identified as *Klebsiella pneumoniae* strain ORWB1 & *Acinetobacter calcoaceticus* strain ORWB3^16^. In the present study, this tertiary consortium was used for development of a sustainable process for production of bio-crude oil using microbial biomass as potential feedstock.

### Characterization of growth and wastewater treatment efficiency of the tertiary consortium on different types of wastewater

With the aim of assessing the performance of the tertiary consortium in terms of biomass concentration and wastewater treatment efficiency, the pH values of all the four wastewater samples were adjusted to the optimal value of 8.6^16^. For TWW and LWW, the concentration of total nitrogen was adjusted to the optimal value of 0.29 g L^−1 ^^16^. The phosphorus concentration of only LWW was adjusted to the optimal value of 49.5 mg L^−1^ as reported by Makut *et al*.^16^. The composition of PWW and MWW was kept unaltered. Thereafter, the wastewater samples were inoculated with 18% (v/v) inoculum in which microalgae and bacteria were extant in the ratio of 1:1^16^. The inoculated wastewater samples were incubated for 7 days in an orbital shaker (Multitron-Pro, Infors HT, Switzerland) at 150 rpm, 30 °C under 100 μE m^−2^ s^−1^ light intensity with a photoperiod of 16:8 h light and dark cycle. All the experiments were carried out in duplicate in 500 mL shake flasks. At the end of the incubation period, the wastewater samples were characterized and screened on the basis of total biomass concentration along with removal efficiency of nutrients and heavy metals. The selected wastewater was then considered for subsequent process development. Dynamic profiles for growth of microalgae, utilization of total nitrogen, phosphate and COD were obtained by analyzing the samples at every 24 h interval. The total biomass (microalgae plus bacteria) concentration achieved at the end of each batch was determined in terms of dry cell weight (DCW). Heavy metal removal efficiency was calculated by analyzing the fermentation broth at the end of the cultivation. The removal efficiency of total nitrogen, phosphate, COD and heavy metals was calculated using Eq. (1):

1 $$Removal\,efficiency\,( \% )=(\frac{{S}_{i}-{S}_{f}}{{S}_{i}})\times 100$$

where, *S*_*i*_ and *S*_*f*_ are the concentrations of a specific nutrient or heavy metal before and after cultivation respectively.

### Batch and fed-batch mode of cultivation of tertiary consortium in automated bioreactor utilizing paper industry wastewater

Based on the characterization of tertiary consortium on different wastewater samples (detailed in the previous section), PWW was selected as the best culturing medium supporting maximum growth and nutrient removal efficiency. With the aim of evaluating the performance of tertiary consortium on PWW at a larger scale of operation, further experiment was performed in a 7.5 L automated photobioreactor (Biojenik Engineering, India) with a working volume of 4 L in batch mode of cultivation wherein the wastewater was inoculated (inoculum size of 18%, v/v) with (i) two bacteria only (designated as bacterial batch, BB); (ii) two microalgae only (designated as microalgal batch, MB) and (iii) two microalgae & two bacteria (designated as tertiary consortium batch, TCB) present in the tertiary consortium. The photobioreactor was operated at 30 °C with an agitation of 150 rpm, aeration and light intensity being 1 vvm and 250 μE m^−2^ s^−1^ respectively for a light:dark cycle of 16:8 h. The BB was conducted for 5 days while the MB and the TCB were run for 10 days. For all the three batches, the initial pH value, total nitrogen and phosphate concentrations were adjusted to their corresponding optimal values as detailed by Makut *et al*.^16^. Sampling was carried out at regular intervals of 24 h for the analysis of total nitrogen, phosphate and COD. Dynamic profiles for growth of microalgae (in case of MB and TCB) and bacteria (only for BB) were obtained by measuring chlorophyll-a content and DCW respectively. Final microbial biomass (microalgae plus bacteria) concentration in terms of DCW was measured at the end of each batch. Dynamic profiles of dissolved oxygen (dO_2_) and dissolved carbon dioxide (dCO_2_) concentrations were obtained for all the three individual batches. Removal efficiency of the nutrients and heavy metals was estimated as described before.

In the next step, a fed-batch strategy was implemented to maximize the microbial biomass concentration. The fed-batch was operated for a period of 18 days under same cultivation conditions as detailed for the batch mode. An intermittent feeding of the key nutrients, nitrogen source and phosphate, was performed in order to maintain their concentrations at their respective optimal values of 0.29 g L^−1^ and 49.5 mg L^−1^ throughout the entire course of fermentation. The concentrations of these key nutrients in the culture broth were monitored every 8 h. The growth of microalgae (in terms of chlorophyll-a content) and COD were estimated every 24 h. At the end of the experiment, the biomass was collected, centrifuged at 8000 rpm and processed for HTL. The total biomass (microalgae plus bacteria) concentration achieved was determined in terms of DCW.

### Analysis of growth, nutrient utilization, COD and heavy metal ion removal

Chlorophyll-a was estimated following the protocol described by Makut *et al*.^16^. The total nitrogen content was determined as the summation of the individual concentrations of nitrate, nitrite and ammoniacal nitrogen. Estimation of nitrate in the supernatant was carried out using the salicylic acid method with sodium nitrate as the standard^40^. The mole fraction and the corresponding nitrogen concentration in nitrite and ammonia was measured and calculated according to the Standard Methods for Examination of Water and Wastewater^41^. Phosphate concentration was estimated using the ascorbic acid method with potassium hydrogen phosphate (dibasic) as standard^42^. The COD was analyzed with HACH COD reagents and quantified in DR900 colorimeter (Hach, USA). For determining the heavy metal concentration, the samples were centrifuged at 13,000 rpm for 5 min and the clear supernatant was analyzed using atomic absorption spectroscopy (Varian AA240, Australia). Standard procedures^41,43^ were followed for the analysis. All chemicals and reagents were procured from Hi Media, India and were of analytical grade.

### Hydrothermal liquefaction of microbial biomass and sample preparation

HTL experiments were carried out for all three types of biomass feedstock obtained from TCB, MB and BB. The HTL experiment was performed using a stainless steel stirred tank batch reactor (Amar Equipments Pvt. Ltd., India) with a capacity of 750 mL. The biomass loading was kept at 15% (w/v) of the total working volume of 200 mL. The reaction temperature was maintained at 310 °C for 55 min at corresponding pressure with a heating rate of 10 °C min^−1 ^^35^. The reaction residence time was calculated from the time point the reactor reached the desired temperature. Supply of heat to the reactor was stopped on attaining the desired holding time. Dichloromethane (DCM) (99%, Hi-media) was added into the reactor once the reaction was complete and subsequently cooled, in order to separate the reaction mixture into the water soluble phase and DCM soluble phase. Then this mixture was filtered using Whatman ash less grade-41 filter paper (20 µm) to separate the biochar. The extraction and separation of the organic phase and the aqueous phase was successively carried out using DCM in the separating funnel. The solvent from the organic phase was removed by evaporation under vacuum in a rota evaporator unit (R-300 digital, Buchi, Switzerland) at 60 °C to obtain the bio-crude oil. The bio-crude yield was calculated using the following Eq. (2):

2 $$Yield\,of\,bio-crude\,oil\,(wt.\, \% )=\frac{Weight\,of\,the\,bio-crude}{Weight\,of\,the\,dry\,microbial\,biomass}\times 100$$

### Analysis of bio-crude oil

#### Gas chromatography-Mass spectrometry

The composition of the bio-crude oil was analyzed by Gas chromatography-Mass spectrometry (GC-MS) (Agilent Technologies, USA) using HP5-MS capillary column (30 m, 0.25 mm id, 0.25 mm film thickness). The inlet temperature and split ratio were maintained at 300 °C and 20:1 respectively. 2 µL of the sample was injected into the GC–MS system consisting of an Agilent 7890B gas chromatograph and Agilent 5977B mass selective detector. The temperature of the column was initially held at 50 °C for 5 min and then ramped to 300 °C at a rate of 10 °C min^−1^. On attaining 300 °C, the temperature was maintained isothermally for 4 min, thereby amounting to a total run time of 37 min. Helium was used as the carrier gas with a constant flow rate 1.6 mL min^−1^. Data acquisition of the chromatogram peaks was carried out using the Mass Hunter WorkStation and the probable compounds were identified using NIST Mass Spectral Database (NIST 14).

#### Thermogravimetric analysis

Thermogravimetric analysis (TGA) of the bio-crude oil was performed using a thermogravimetric analyser (STA7200, Hitachi, Japan) in an inert atmosphere of nitrogen. The flow rate of nitrogen was maintained at 50 mL min^−1^. For TGA analysis of the bio-crude oil, 10 µL of the sample was taken in the alumina crucible and heated from room temperature to 700 °C at a heating rate of 20 °C min^−1^. The sample weight loss with respect to rise in temperature was recorded to estimate the boiling point distribution.

#### Fourier transform infrared spectroscopy

Fourier transform infrared spectroscopy (FTIR) analysis of the bio-crude oil was conducted using IR-Affinity-1 (Shimadzu, Japan) in order to analyze the functional groups present in the sample. The infrared spectrum range was between 400 cm^−1^ and 4000 cm^−1^ and scanning was executed at the rate of 20 with a step size of 4 cm^−1^.

### Ethical approval

This article does not contain any studies with human participants or animals performed by any of the authors.

## Supplementary information

Supplementary File

## Data Availability

The data that support the findings of this study are available on request from the corresponding author.
