# Supplementary material for: Sustainable production of bio-crude oil via hydrothermal liquefaction of symbiotically grown biomass of microalgae-bacteria coupled with effective wastewater treatment
Source: Sci Rep. 2019 Oct 18;9:15016. doi: 10.1038/s41598-019-51315-5 (PMC6802377; doi:10.1038/s41598-019-51315-5)
Supplement: Supplementary file 1 — Supplementary File [file 41598_2019_51315_MOESM1_ESM.pdf]

SUPPLEMENTARY FILE

Sustainable production of bio-crude oil via hydrothermal liquefaction of symbiotically grown biomass of microalgae-bacteria coupled with effective wastewater treatment

Gargi Goswami[a,¥], Bidhu Bhusan Makut[b,¥], Debasish Das[a,b,*]

[a]*Department of Biosciences & Bioengineering, Indian Institute of Technology, Guwahati, Assam 781039, India*

[b]*Center for Energy, Indian Institute of Technology, Guwahati, Assam 781039, India*

[¥]Equal author contribution

*Corresponding author.

Department of Biosciences & Bioengineering,

Indian Institute of Technology Guwahati,

Guwahati, Assam 781039, India.

Tel.: +91-361 258 2221; fax: +91-361 258 2249.

E-mail addresses: debasishd@iitg.ac.in, debasis.iitb@gmail.com (D. Das).

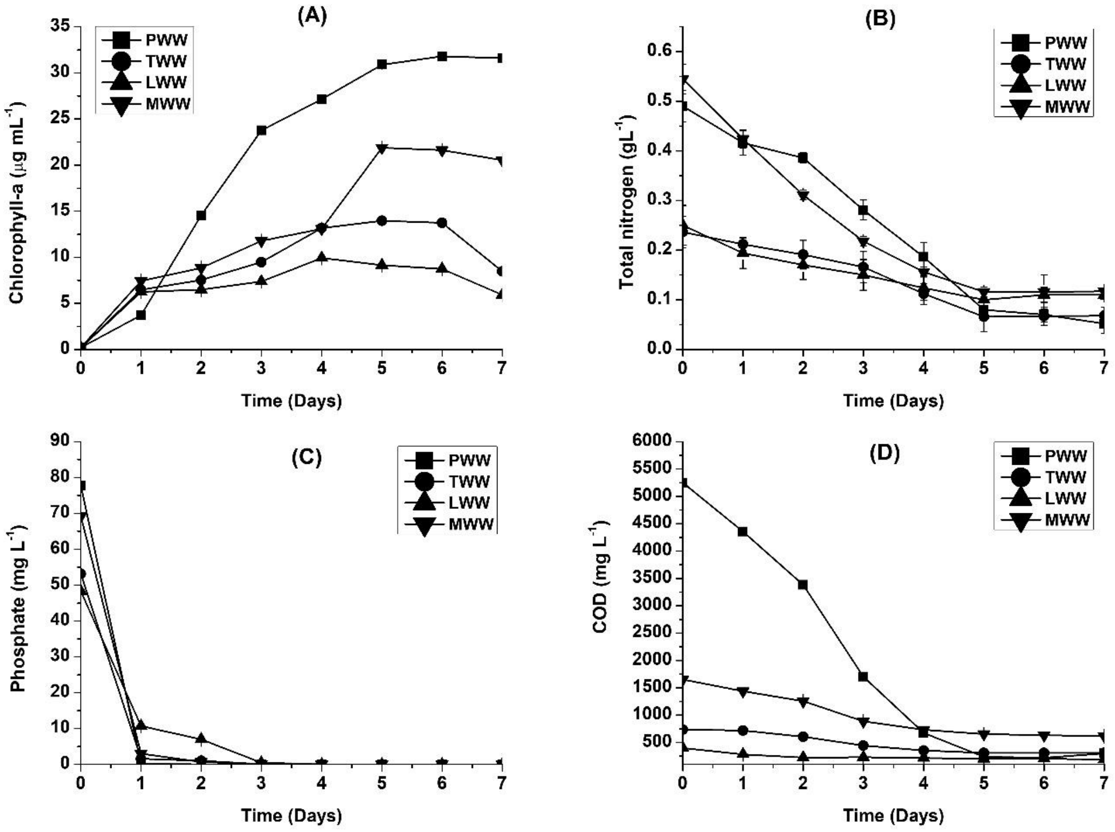

Supplementary Figure S1. Dynamic profiles for (A) growth of microalgae (Chlorophyll-a, μg mL^{-1}); (B) total nitrogen (g L^{-1}); (C) phosphate (mg L^{-1}) and (D) COD (mg L^{-1}) of the tertiary consortium grown on paper industry wastewater (PWW), textile industry wastewater (TWW), leather industry wastewater (LWW) and municipal wastewater (MWW).

Supplementary Table S1. Detailed material balance for total nitrogen, phosphate and COD present in different types of wastewater.

Basis: 1 L of wastewater

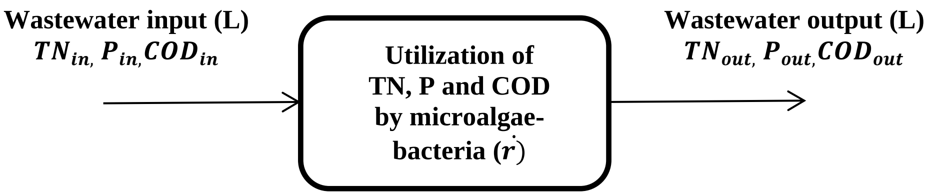

TN: Total Nitrogen, P: Phosphate and COD: Chemical Oxygen Demand

(A)

Nutrient	Wastewater type: Paper Industry Wastewater (PWW)					
	Input (mg)	Generation (mg)	Output (mg)	Consumption (mg)	Accumulation (mg)	% removal
TN	0.49	0	0.05	0.44	0	89.3
P	77.7	0	0	77.7	0	100
COD	5250	0	300	4950	0	94.23

(B)

Nutrient	Wastewater type: Textile Industry Wastewater (TWW)					
	Input (mg)	Generation (mg)	Output (mg)	Consumption (mg)	Accumulation (mg)	% removal
TN	0.23	0	0.067	0.163	0	70.99
P	53.16	0	0	53.16	0	100
COD	735	0	299.3	435.7	0	59.28

(C)

Nutrient	Wastewater type: Leather Industry Wastewater (LWW)					
	Input (mg)	Generation (mg)	Output (mg)	Consumption (mg)	Accumulation (mg)	% removal
TN	0.25	0	0.111	0.139	0	55.66
P	48.3	0	0	48.3	0	100
COD	400.5	0	189.23	211.26	0	52.75

(D)

Nutrient	Wastewater type: Municipal Wastewater (MWW)					
	Input (mg)	Generation (mg)	Output (mg)	Consumption (mg)	Accumulation (mg)	% removal
TN	0.54	0	0.116	0.423	0	78.48
P	69.2	0	0	69.2	0	100
COD	1650	0	617.1	1032.9	0	62.6
